# Radiation-Induced Oxidative Stress at Out-of-Field Lung Tissues after Pelvis Irradiation in Rats

**DOI:** 10.22074/cellj.2016.4561

**Published:** 2016-08-24

**Authors:** Masoud Najafi, Reza Fardid, Mohammad Ali Takhshid, Mohammad Amin Mosleh-Shirazi, Abol-Hassan Rezaeyan, Ashkan Salajegheh

**Affiliations:** 1 Department of Radiology, School of Paramedical Sciences, Shiraz University of Medical Sciences, Shiraz, Iran; 2 Department of Medical Physics, School of Medicine, Tehran University of Medical Sciences, Tehran, Iran; 3 Diagnostic Laboratory Sciences and Technology Research Center, School of Paramedical Sciences, Shiraz University of Medical Sciences, Shiraz, Iran; 4 Department of Radiotherapy, School of Medicine, Shiraz University of Medical Sciences, Shiraz, Iran

**Keywords:** Bystander Effect, Out of Field, Radiation, Lung, Oxidative Stress

## Abstract

**Objective:**

The out-of-field/non-target effect is one of the most important phenomena of ionizing radiation that leads to molecular and cellular damage to distant non-irradiated tissues. The most important concern about this phenomenon is carcinogenesis many years after radiation treatment. *In vivo* mechanisms and consequences of this phenomenon are not known completely. Therefore, this study aimed to evaluate the oxidative damages to out-of-field lung tissues 24 and 72 hours after pelvic irradiation in rats.

**Materials and Methods:**

In this experimentalinterventional study, Sprague-Dawleymale rats (n=49) were divided into seven groups (n=7/each group), including two groups of pelvis- exposed rats (out-of-field groups), two groups of whole bodyexposed rats (scatter groups), two groups of lung-exposed rats (direct irradiation groups), and one control sham group. Out- of-field groups were irradiated at a 2×2 cm area in the pelvis region with 3 Gy using 1.25 MeV cobalt-60 gamma-ray source, and subsequently, malondialdehyde (MDA) and glutathione (GSH) levels as well as superoxide dismutase (SOD) activity in out-of-field lung tissues were measured. Results were compared to direct irradiation, control and scatter groups at 24 and 72 hours after exposure. Data were analyzed using Mann-Whitney U test.

**Results:**

SOD activity decreased in out-of-field lung tissue 24 and 72 hours after irradiation as compared with the controls and scatter groups. GSH level decreased 24 hours after exposure and increased 72 hours after exposure in the out-of-field groups as compared with the scatter groups. MDA level in out-of-field groups only increased 24 hours after irradiation.

**Conclusion:**

Pelvis irradiation induced oxidative damage in distant lung tissue that led to a dramatic decrease in SOD activity. This oxidative stress was remarkable, but it was less durable as compared to direct irradiation.

## Introduction

The non-target effect is one of important phenomena of ionizing radiation that leads to molecular and cellular damage to distant non-irradiated tissues. The most important concern about this phenomenon is carcinogenesis many years after radiation treatment that most likely depends on irradiated cells and receptor cells of bystander signals. *In vivo* studies have shown that this is a tissuespecific phenomenon (1) and indicated different irradiated tissues cause changes in distant tissues (2). Several reviews have confirmed that lung cancer is one of the most important cancers with high incidence among patients undergoing radiation treatment for cervical, ovarian, rectal and prostate cancers (3,6). It has been also proposed that second primary lung cancer among these people is mainly attributed to out-of-field effect, not direct radiation exposure (7). 

The several factors involved in radiation-induced non-target effects can produce free radicals and nitric oxide, including nicotinamide adenine dinucleotide phosphate (NADPH) oxidase, inducible nitric oxide synthetize (iNOS), and cyclooxygenase-2 (COX-2). NADPH oxidase as a membrane-boundenzyme complex is used by neutrophils to destroy pathogens (8). Furthermore, NADPH oxidase activated by protein kinases, p38 and transforming growth factor-beta (TGF-β) can consistently produce free radicals after irradiation exposure (9). A study on fibroblasts and epithelial cell lines has suggested that this enzyme plays a key role in the bystander effect by continuous production of free radicals (10). *iNOS* gene is expressed by macrophages that are activated by increased production of cytokines such as interleukin-1 (IL-1), IL-2, IL-6, IL-8, TGF-β and tumor necrosis factor (TNF-α) that subsequently stimulate the production of nitric oxide, leading to increased chromosomal damage, changes in the gene expression, mutagenesis, and apoptosis in non-irradiated cells (10). In addition to *iNOS*, these cytokines can also increase the production of *COX-2* enzyme. *COX-2* gene is expressed specifically in some tissues such as the lung, heart and liver. *In vitro* and *in vivo* studies have shown *COX-2* is one of the most important factors involved in radiation-induced by stander effect and reported three-fold increase of *COX-2* level (11). Chai et al. (12) also have shown 20-fold increase of *COX-2* level in bystander lung tissue 24 hours after irradiation of the abdomen. They have also suggested that *COX-2* is a very important factor in causing cell damage due to out-of-field effect. 

Another important factor contributing to oxidative damage caused bystander effect is the mitochondria that act as a free radical generating agent (13). A number of studies have indicated the following factors decreasing the chromosome damages in bystander cells: lack of cytochrome c, suppression of mitochondrial respiratory chain, as well as presence of cells lacking mitochondrial DNA (mtDNA) (14,15). Zhou et al. (16) have compared cells lacking mtDNA with normal cells and showed that the absence of mitochondria reduced mutagenesis in bystander cells through inhibiting the expression of other genes involved in this process such as *COX-2* and *iNOS*. Since lung cancer is one of most common cancer after pelvis radiotherapy; therefore, this study aimed to evaluate oxidative damage to out-of-field lung tissue 24 and 72 hours after pelvic irradiation in rats using the measurement of the malondialdehyde (MDA) and glutathione (GSH) levels as well as superoxide dismutase (SOD) activity. 

## Materials and Methods

In this experimentalinterventional study, 49 male Sprague-Dawley rats were purchased from the Center of Comparative and Experimental Medicine, Shiraz University of Medical Sciences, Shiraz, Iran. Rats were housed in the Animal House of Shiraz University of Medical Sciences. All the steps in this regard were taken in accordance with the Guide for the Care and Use of Laboratory Animals of Shiraz University of Medical Sciences. All animals were kept under controlled conditions of temperature, humidity and light. Sprague-Dawley male rats were divided into following seven groups (n=7/each group): two groups of pelvisexposed rats (out-of-field groups) receiving a pelvic exposure to 3 Gy gamma rays and other tissues were protected by lead shield, two groups of whole body-exposed rats (scatter groups) receiving a whole-body exposure to 7.5 mGy, two groups of lung-exposed rats (direct irradiation) receiving a lung exposure to 3 Gy gamma rays, and one control sham group receiving no exposure. MDA and GSH levels as well as SOD activity were measured 24 and 72 hours after irradiation using 1.25 MeV cobalt-60 γ (gamma)-ray source. Prior to irradiation, the rats received anesthesia using a combination of ketamine (50 mg/kg) and xylazine (10 mg/ kg) via an intramuscular injection. Subsequently, the animals were sacrificed, and lung tissues were extracted and frozen at -80˚C. 

### Irradiation and measurement of scattering radiation dose

The lung and pelvis were exposed to 3 Gy at a dose rate of 30 cGy/minute with a 1.25 MeV cobalt-60 γ-radiation source. Local irradiation was performed at a 2×2 cm area of the animal’s pelvis. A plexiglas rat phantom was used for measurement of scattering radiation dose received by lung. The semiflex ionization chamber was also used to measure the scattering dose in distant rat phantom lung tissue. The phantom was prepared from Plexiglas layers and cork as a lung-soft tissue equivalent. The measured radiation dose in lung-tissue equivalent after irradiation of the pelvis in rat phantom with 3 Gy of γ-ray dose was 7.5 mGy. This radiation dose was received by the scatter groups. 

### Glutathione assay and superoxide dismutase activity assays

For GSH assay, the lung tissues were homogenized in 0.1 mM cold phosphate buffered saline (PBS, PH=7.0). For SOD assay, lung tissues were homogenized in cold 20 mM 4-(2-hydroxyethyl)1-piperazineethanesulfonic acid (HEPES, PH=7.2) buffer (Sigma Aldrich), containing 1 m Methylene glycol tetra acetic acid (EGTA), 210 mM mannitol, and 70 mM sucrose (Sigma Aldrich) per gram tissue. Samples were centrifuged and supernatants were collected. GSH and SOD assays were performed by GSH and SOD assay kits (Cayman Chemical, USA). 

### Malondialdehyde assay

Thiobarbituricacid (TBA), trichloroacetic acid (TCA) and 1,1,3,3tetraethoxypropane (TEP) were purchased from Sigma Aldrich, Germany. The MDA level was used as the product of oxidative damage to cells. The lung tissue samples were homogenized in PBS buffer (pH=7.0) and centrifuged at 5000 RPM for 5 minutes. Supernatants were collected and the level of MDA was measured by spectrophotometric method. The colored product of lipid peroxidation with TBA-TCA reagent was prepared and the absorbance was read at 532 nm. MDA standard was prepared by dissolving 25 µL TEP. 

### Statically analysis

The mean ± SD was calculated and statistical analysis was done using the Statistical Package for the Social Sciences (SPSS, SPSS Inc., USA) version 16. Data were analyzed using Mann-Whitney U test to determine the significance of the mean differences. P values lower than 0.05 were considered significant. 

## Results

### Superoxide dismutase activity

**Fig.1:**
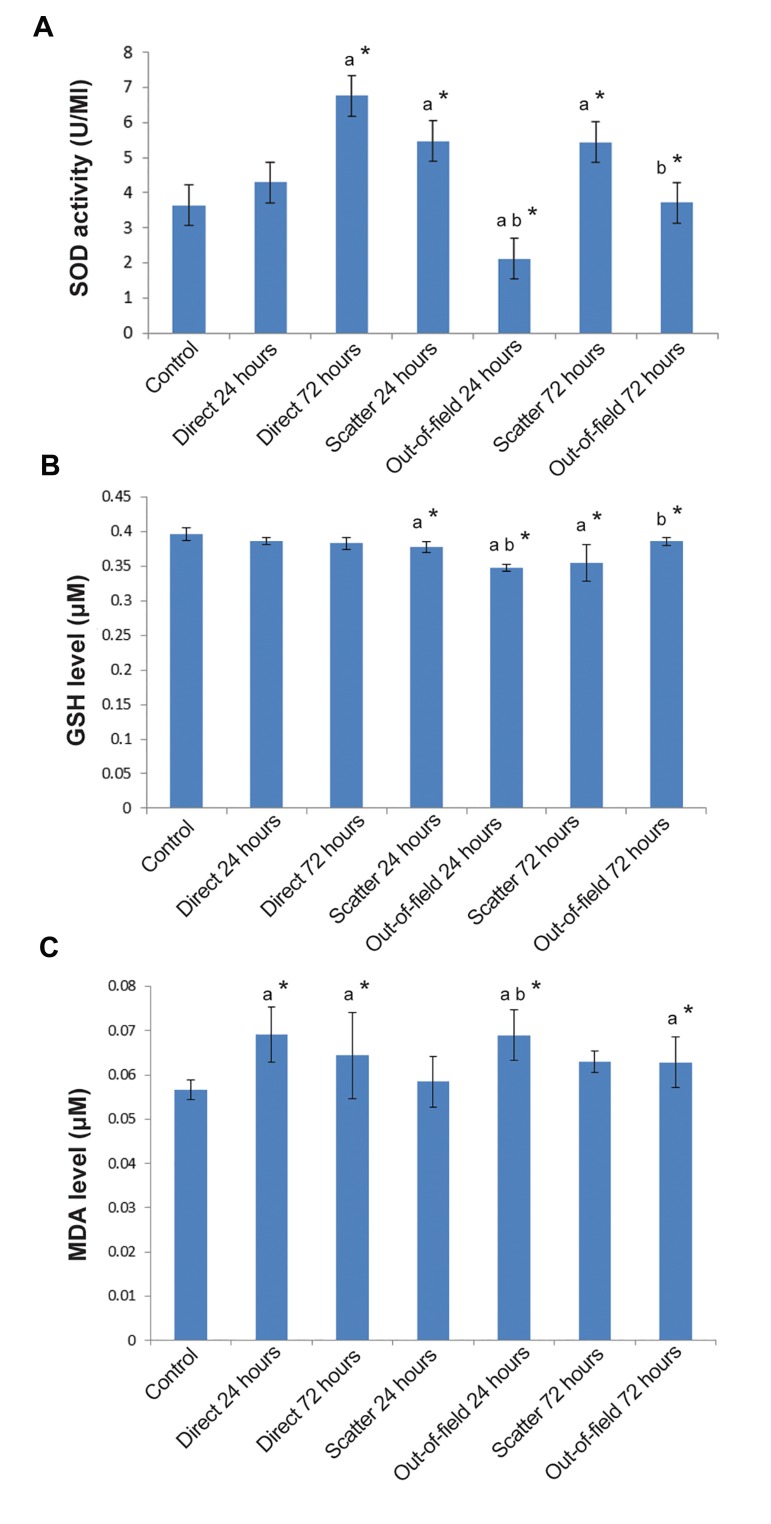
Changes in oxidative stress markers include SOD activity, GSH and MDA levels in lung tissue. A. SOD activity, B.GSH level, and C. MDA level in the lung tissues in the direct, scatter and out-of-field groups. Direct irradiation and scatter groups were compared to the control group, while out-of-field groups were compared to the scatter groups. a; Values are expressed as a comparison between treatment and control groups, b; Out- of-field groups are compared with scatter groups using Mann- Whitney test, *; P<0.05, SOD; Superoxide dismutase, GSH; Glutathione, and MDA; Malondialdehyde.

Direct irradiation led to a significant increase in SOD activity at 72 hours (6.766 ± 1.08 U/ml) after irradiation (P<0.05), but nothing was detected 24 hours after irradiation (4.295 ± 1/09 U/ml), in comparison to the normal control group (3.634 ± 0.66 U/ml). In the out-of-field group, 24 hours after exposure, SOD activity significantly decreased (2.11 ± 0.58 U/ml) in comparison to the related value of scatter groups (5.47 ± 0.85 U/ml). Furthermore, 72 hours after exposure, a significant decrease in SOD activity was observed in the outof-field group (3.72 ± 0.58 U/ml) in comparison to the related value of scatter groups (5.43 ± 1.001 U/ ml). Scatter dose increased the SOD activity 24 and 72 hours after irradiation as compared to the normal control (P<0.05) (Fig.1A). 

### Glutathione

Irradiation to the lung tissue showed no significant decrease in GSH level 24 (0.387 ± 0.005 μM) and 72 hours (0.383 ± 0.009 μM) after irradiation as compared with the control group (0.396 ± 0.009 μM). The out-of-field irradiation led to a significant decrease (P<0.05) in GSH level 24 hours after exposure (0.348 ± 0.005 μM) as compared to the related value of scatter groups (0.378 ± 0.008 μM), whereas 72 hours after exposure, there was a significant elevation (P<0.05) in GSH level (0.386 ± 0.006 μM) as compared to the related value of the scatter groups (0.355 ± 0.027 μM). Scattered radiation resulted in a decrease in GSH level at both 24 and 72 hours after irradiation (P<0.05) (Fig.1B). 

### Malondialdehyde

The MDA level elevated 24 (0.0691 ± 0.0063 μM) and 72 hours (0.0644 ± 0.0097 μM) after direct irradiation as compared to the control group (0.0567 ± 0.0027 μM). These increases were significant at both 24 and 72 hours after exposure (P<0.05). The out-of field effect could increase MDA level only 24 hours after irradiation (0.0690 ± 0.0053 μM) as compared to the related values of scatter (0.0585 ± 0.0057 μM) and control groups (P<0.05). However, out-of-field effect failed to elevate MDA level 72 hours after exposure (0.0628 ± 0.0057 μM) as compared to the related value of scatter groups. The MDA level significantly increased in the scatter group 72 hours after irradiation (0.0630 ± 0.0025 μM) as compared to the control group (P<0.05), indicating there was no significant difference regarding MDA level 24 hours after irradiation between the scatter and the control groups (Fig.1C). 

## Discussion

In this study, we described a significant oxidative damage that occurred in rat lung after lung and pelvis irradiation. The main findings of this study were oxidative damage induced by out-of-field effect similar to direct irradiation and our results revealed that out-of-field effect changed the SOD activity and GSH level. Furthermore, the scatter groups demonstrated that the changes in the outof-field lung tissues were not caused by the scattered radiation. The *in vivo* and *in vitro* studies have suggested that this phenomenon has important biologic consequences within non-irradiated cells and tissues. The previous studies have also showed that immune signaling and epigenetic modulators are the most important factors involved in distant tissue damages after local irradiation (7). Calveley et al. (17) have reported that out-of-field effect increases inflammatory cytokines such as IL-1, IL-6, TNF-α and TGF-β in lung tissue. Immune cells, such as macrophages and lymphocytes, have an important role in chronic inflammation and oxidative damages in lung tissue. These immune cells by increasing reactive oxygen species (ROS) and NO production after up-regulation of the inflammatory cytokines lead to increased mutagenesis and inflammatory responses in non-irradiated cells (18). Production of pro-inflammatory cytokines and ROS generation can related to induction of genomic instability in both irradiated and out-of-field tissues (19). 

Epigenetic effectors such as micro-RNAs are other important factors that may contribute in ROS production and inhibition of SOD activity in nonirradiated tissues. *In vitro*/ study has also shown the role of miR-21 in radiation-induced bystander effect (20). The TGF-β causes an increase in ROS levels through miR-21in bystander cells (21). Furthermore, reduced SOD activity may be related to epigenetic changes (22). Some studies have indicated that lung cancer is one of most common secondary cancer resulted of radiation therapy to pelvic area such as prostate (3,23,24), ovarian (5) and rectal cancer (6). It seems oxidative damages induced by out-of-field effect can be involved in the high incidence of lung cancer in these patients. Although it is thought that high incidence of lung cancer among these patients is unlikely due to the scattered radiation. 

In this study, we used a 3 Gy single dose of γ-radiation and evaluated increased oxidative stress in out-of-field and direct irradiated lung tissues at 24 and 72 hours after irradiation. Direct irradiation led to an increase in MDA level at both 24 and 72 hours after exposure toγ-radiation. Our findings showed that GSH level in out-of-field lung tissues and animals exposed to scattered radiation (7/5 mGy) decreased at both 24 and 72 hours after exposure, whereas direct irradiation by higher dose (3Gy) failed to decrease GSH levels at the same time period. Furthermore, SOD activity increased at 72 hours as compared to 24 hours in both direct irradiation and out-of-field groups, but not in scatter radiation groups. We showed that pelvis irradiation with a single 3 Gy γ-radiation induced oxidative damage in the distant lung tissue, including an increase in MDA level, a slight decrease in GSH level, and a dramatic decrease in SOD activity. Our findings also demonstrated that the changes in oxidative stress levels in out-of-field lung tissue were not durable, and the changes in MDA level at 72 hours after exposure were significant as compared to scatter group. According to these results, it seems that inhibition of SOD activity for several days was a major effect of out-of-field effect, leading to increased oxidative damages. However, direct irradiation did not cause inhibition of SOD activity. Our results revealed that important changes were associated with out-of-field effect, occurring on the first day after irradiation. 

## Conclusion

We evaluated oxidative stress by a single radiation dose. During conventional radiotherapy, patients undergo fractionated treatments, 5 days per week for 7 weeks. In these conditions, increased level of MDA and decreased activity of SOD result in increasing genomic instability and cancer risk due to oxidative damage induced by out-offield effect in long-term cancer survivors. It seems production of ROS and suppression of antioxidant enzymes activity are the most important causes of oxidative damages at out-of-field organs. Further studies are needed to explore different signaling pathways involved in ROS production by this phenomenon. 
